# Nonclassical Congenital Adrenal Hyperplasia Presenting With Isolated Hirsutism in a Young Adult: A Case Report

**DOI:** 10.7759/cureus.88750

**Published:** 2025-07-25

**Authors:** Kasra Navabi, Vivek Bose

**Affiliations:** 1 Endocrinology, Diabetes and Metabolism, University of California Los Angeles David Geffen School of Medicine, Los Angeles, USA

**Keywords:** 21-hydroxylase deficiency, acth stimulation test, adrenal steroidogenesis, cyp21a2 mutation, hirsutism, hyperandrogenism, isolated hirsutism, late-onset cah, nccah, nonclassical congenital adrenal hyperplasia

## Abstract

Congenital adrenal hyperplasia (CAH) comprises a group of autosomal recessive disorders caused by enzymatic deficiencies in adrenal steroidogenesis, resulting in impaired cortisol biosynthesis. These deficiencies may manifest as ambiguous genitalia in neonates, salt-wasting crises, or hirsutism in women. Although CAH is typically diagnosed in infancy or early childhood, nonclassical (late-onset) forms may present later in life. Nonclassical congenital adrenal hyperplasia (NCCAH), a milder variant of 21-hydroxylase deficiency, often manifests during adolescence or adulthood and remains an underrecognized cause of hyperandrogenism in females. We report the case of a 21-year-old woman who presented with isolated hirsutism in the absence of virilization or menstrual irregularities. Laboratory evaluation revealed elevated 17-hydroxyprogesterone and androgens, and the diagnosis was confirmed with an adrenocorticotropic hormone (ACTH) stimulation test. Imaging studies were unremarkable. Treatment was deferred due to the patient’s regular menstrual cycles and her desire for fertility. This case underscores the importance of hormonal evaluation in isolated hirsutism and the role of ACTH stimulation testing in the definitive diagnosis of NCCAH.

## Introduction

Congenital adrenal hyperplasia (CAH) is a group of autosomal recessive disorders caused by defects in cortisol biosynthesis. Nonclassical congenital adrenal hyperplasia (NCCAH), the milder and more prevalent late-onset form, typically results from partial 21-hydroxylase deficiency caused by mutations in the CYP21A2 gene. Unlike classic CAH, NCCAH is not life-threatening and often remains undiagnosed until adolescence or adulthood. It most commonly presents with signs of androgen excess, such as hirsutism, acne, and/or oligomenorrhea. NCCAH is frequently underrecognized and may be misdiagnosed as polycystic ovary syndrome (PCOS) or idiopathic hirsutism [1].

## Case presentation

A 21-year-old woman presented with progressive hirsutism since the age of 16, involving the upper lip, chin, chest, abdomen, back, arms, and thighs. She had undergone several sessions of laser hair removal. She denied voice deepening, clitoromegaly, weight changes, or menstrual irregularities. She reported no medication use. Past medical and family history were unremarkable. She was a non-smoker, abstained from alcohol and recreational drugs, and had regular menstrual cycles and ultimately experienced a successful pregnancy, resulting in the delivery of a healthy child.

**Figure 1:**
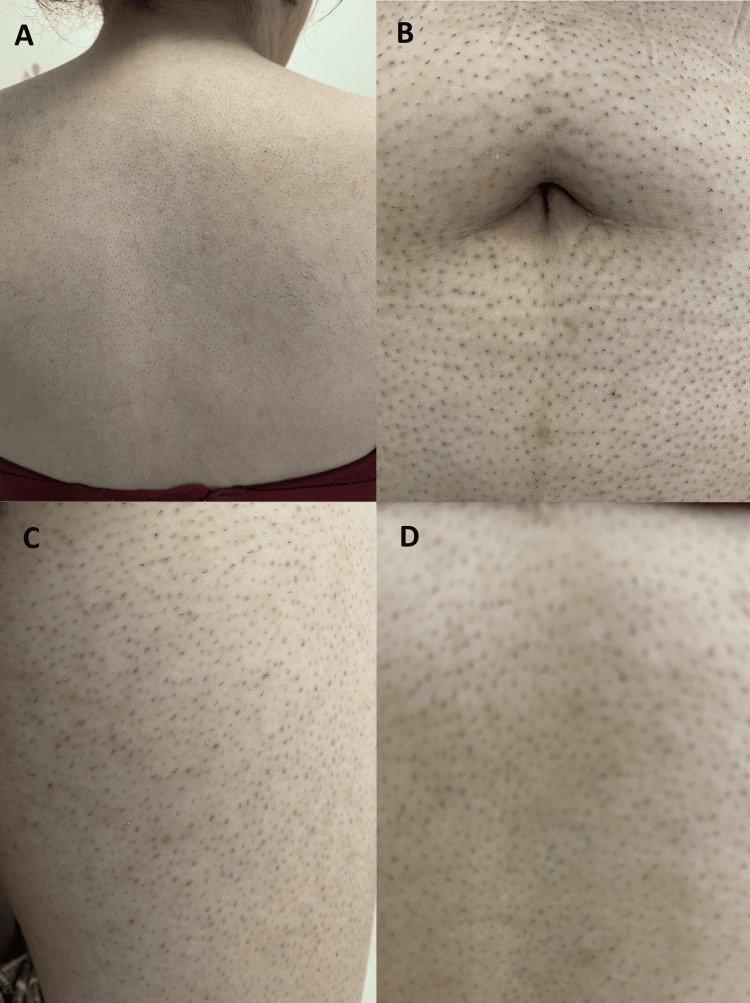
Hirsutism involving the (A) back, (B) abdomen, and (C, D) extremities (coarse hair follicles after shaving)

On physical examination, vital signs were normal. Coarse, terminal hair was noted on the upper lip, chin, midsternal chest, abdomen, upper arms, back, inner thighs, and buttocks (Figure 1).

Initial laboratory studies demonstrated elevated androgens and 17-hydroxyprogesterone (17-OHP), with a high dehydroepiandrosterone sulfate (DHEA-S) level (Table 1). An ACTH stimulation test was performed using 250 µg of intravenous (IV) cosyntropin (Table 2). A stimulated 17-OHP level > 1000 ng/dL confirms the diagnosis of NCCAH [2]. Abdominal and pelvic computer tomography (CT) scans did not show any adrenal mass or nodule, and her bilateral ovaries were normal, without any mass or cysts. At the same time, a pelvic ultrasound confirmed normal ovaries and adnexa and was not in favor of polycystic ovaries.

**Table 1:** Laboratory findings TSH: thyroid-stimulating hormone, T4: thyroxine, DHEA-S: dehydroepiandrosterone sulfate, FSH: follicle-stimulating hormone, LH: luteinizing hormone.

Laboratory findings	Result	Reference range
Sex hormone-binding globulin	8 nmol/L, low	30-135 nmol/L
Testosterone, total	42 ng/dL	9-55 ng/dL
Testosterone, free	10.9 pg/mL, high	0.8-7.4 pg/mL
Testosterone, bioavailable	33.6 ng/dL, high	2.2-20.6 ng/dL
TSH	1.3 mcIU/mL	0.3-4.7 mcIU/mL
Free T4	1.3 ng/dL	0.8-1.7 ng/dL
17-Alpha-hydroxyprogesterone	579.56 ng/dL, high	≤206.00 ng/dL
DHEA-S	1170 mcg/dL, high	40-360 mcg/dL
Estradiol	98 pg/mL	20-400 pg/mL
FSH	4.8 mIU/mL	6-23 mIU/mL
LH	12.5 mIU/mL	1-91 mIU/mL
Prolactin	14.4 ng/mL	3-23.1 ng/mL

**Table 2:** ACTH stimulation test results ACTH: adrenocorticotropic hormone.

Time	17-Hydroxyprogesterone		Cortisol		ACTH		DHEA-S	
	Result	Reference range	Result	Reference range	Result	Reference range	Result	Reference range
0 minute	522.32 ng/dL	≤206.00 ng/dL	9 mcg/dL	7-10 mcg/dL	19 pg/mL	7.2-63.3 pg/mL	737 mcg/dL	40-360 mcg/dL
30 minutes	6470.88 ng/dL	<1000 ng/dL	14 mcg/dL	>18 mcg/dL	-		-	
60 minutes	6018.76 ng/dL	<1000 ng/dL	17 mcg/dL	>18 mcg/dL	-		-	

## Discussion

Hirsutism is defined as the presence of terminal coarse hairs in a male-pattern distribution, affecting approximately 5%-10% of women [3]. It is important to distinguish hirsutism from hypertrichosis, which is generalized excessive growth of vellus hair that may be hereditary or result from certain medications (e.g., glucocorticoids, phenytoin, minoxidil, or cyclosporine). Hypertrichosis is not caused by hyperandrogenism.

Once hirsutism is identified, a structured diagnostic evaluation is essential to determine its underlying cause. Significant hirsutism score, typically defined by a modified Ferriman-Gallwey score (mFG score) of 8 or higher. This scoring system assesses terminal hair growth in nine androgen-sensitive areas of the body, assigning a score from 0 (no hair) to 4 (frankly virile) for each site. A total score of 8 or more is considered abnormal and warrants further endocrine evaluation. The patient's calculated mFG score was around 30, which was well above the threshold of ≥8, confirming clinically significant hirsutism [4].

According to the 2018 Endocrine Society Clinical Practice Guidelines, the initial hormonal workup should include measurement of serum total testosterone in all women with a clinically significant hirsutism score. Additionally, serum 17-OHP should be measured to screen for NCCAH, as elevated levels are uniquely associated with this condition [5].

Depending on the clinical presentation and level of suspicion, further hormonal testing may be warranted. DHEA-S levels should be assessed, as markedly elevated values indicate an adrenal source of androgen excess, either benign or malignant. A 24-hour urine-free cortisol test is appropriate in patients with signs suggestive of Cushing’s syndrome, aiding in the detection of cortisol-producing tumors or adrenal hyperplasia. Luteinizing hormone (LH) and follicle-stimulating hormone (FSH) levels can be used to evaluate for polycystic ovary syndrome (PCOS), particularly when the LH/FSH ratio exceeds 3:1 [6]. Prolactin levels should be checked to rule out hyperprolactinemia, which may result from hypothalamic or pituitary disorders and can contribute to hirsutism by increasing adrenal DHEA-S production [7]. Finally, thyroid-stimulating hormone (TSH) levels should be evaluated, as hypothyroidism causes an elevation in thyrotropin-releasing hormone (TRH) and subsequently TSH. Elevation in TRH can also result in hyperprolactinemia; hyperprolactinemia can cause amenorrhea and hirsutism, which can be remediated with treatment of hypothyroidism [5].

The patient did not meet at least two of the three Rotterdam criteria required for a diagnosis of PCOS: (1) she had no ovulatory dysfunction, as evidenced by regular menstrual cycles; (2) imaging via pelvic CT scan and ultrasound did not reveal polycystic ovaries; although (3) she did exhibit hyperandrogenism, manifesting as elevated androgen levels and hirsutism.

Imaging studies are also important to assess for structural causes of hyperandrogenism. Pelvic ultrasonography can detect ovarian neoplasms or polycystic ovarian morphology. In cases where adrenal pathology is suspected, magnetic resonance imaging (MRI) or computed tomography (CT) of the adrenal glands can be performed to evaluate for adrenal tumors or hyperplasia.

In this case, the patient's initial laboratory results confirmed elevations in free and bioavailable testosterone, DHEA-S, and 17-OHP. While serum testosterone can be normal to moderately increased in benign conditions like PCOS and CAH, total testosterone levels > 200 ng/dL should raise concerns for an androgen-secreting neoplasm. The total testosterone level and gradual onset of this patient's hirsutism without signs of virilization made an adrenal or ovarian tumor less likely.

Consistent with the initial assessment, the patient's DHEA-S level was high. The pathophysiology involves partial deficiency of 21-hydroxylase, leading to increased ACTH stimulation and shunting of steroid precursors into the androgen synthesis pathway, resulting in excess adrenal androgens, including DHEAS, androstenedione, and testosterone [8]. Imaging including CT scan did not confirm an adrenal tumor.

Moreover, the hormonal workup was not indicative of hypothyroidism or hyperprolactinemia.

A baseline 17-OHP level < 200 ng/dL effectively excludes a diagnosis of 21-hydroxylase deficiency, whereas a level between 300 and 1,000 ng/dL necessitates an ACTH stimulation test for confirmation as a gold standard test for diagnosis. This patient's baseline 17-OHP was elevated at 579.56 ng/dL. The subsequent ACTH stimulation test demonstrated a post-stimulation 17-OHP level well above the diagnostic threshold of 1,000 ng/dL, confirming the diagnosis of NCCAH. Levels exceeding 1,500 ng/dL post-stimulation are considered definitive, making further genetic testing unnecessary [9]. The biochemical findings in classic 21-hydroxylase deficiency are typically more severe, with basal 17-OHP concentrations often exceeding 3,500 ng/dL [10].

Management of NCCAH is tailored to the patient's symptoms and reproductive goals. For women not seeking immediate fertility, combined oral contraceptives (OCs) are the first-line therapy for managing hyperandrogenic symptoms. Antiandrogens like spironolactone can be added if OCs are insufficient, though their potential for teratogenicity must be considered. Glucocorticoids are another treatment option, used to suppress adrenal androgen production and manage hirsutism. However, due to the potential for significant side effects with long-term use, glucocorticoid therapy is typically reserved for patients who do not respond to or cannot tolerate OCs and antiandrogens [11]. Stress doses of glucocorticoids are not required for patients with NCCAH unless their hypothalamic-pituitary-adrenal axis has been suppressed by ongoing glucocorticoid therapy.

Given that the patient had regular ovulatory cycles and desired future fertility, medical treatment was deferred. She subsequently conceived and successfully delivered a child.

## Conclusions

This case highlights the importance of considering NCCAH in the differential diagnosis of patients presenting with isolated hirsutism, even when menstrual cycles are normal. The ACTH stimulation test remains the gold standard for confirming the diagnosis. Management should be individualized, based on the severity of clinical symptoms and the patient's reproductive goals.
